# Pathologic substrate of gastropathy in Anderson-Fabry disease

**DOI:** 10.1186/s13023-020-01436-2

**Published:** 2020-06-22

**Authors:** Alessandro Di Toro, Nupoor Narula, Lorenzo Giuliani, Monica Concardi, Alexandra Smirnova, Valentina Favalli, Mario Urtis, Costanza Alvisi, Elena Antoniazzi, Eloisa Arbustini

**Affiliations:** 1 Center for Inherited Cardiovascular Diseases, Transplant Research Area, Fondazione IRCCS Policlinico San Matteo, Piazzale Golgi 19, 27100 Pavia, Italy; 2 Division of Cardiology, Department of Medicine, New York Presbyterian Hospital, Weill Cornell Medicine, New York, NY USA; 3 European Institute of Oncology, Department of Experimental Oncology, Milan, Italy; 4 Internal Medicine - Endoscopy Unit, Ospedale Civile di Voghera, Voghera, ASST, Pavia, Italy; 5 Ophthalmology, Fondazione IRCCS Policlinico San Matteo, Pavia, Italy

**Keywords:** Anderson Fabry disease (AFD), Gastropathy, Esophagogastroduodenoscopy (EGD), Globotriaosylceramide (GB3)

## Abstract

In both classic and late-onset AFD, mutations of the GLA gene cause deficient activity of the alpha-galactosidase enzyme resulting in intracellular accumulation of the undigested substrate. Gastrointestinal symptoms (GI) are common but non-specific and imputed to the AFD, irrespective of the demonstration of substrate accumulation in GI cells. We demonstrate substrate accumulation in gastric epithelial, vascular, and nerve cells of patients with classic AFD and, vice versa, absence of accumulation in late-onset AFD and controls.

## Introduction

Anderson Fabry Disease (AFD) (MIM#301500) is a rare (1:40.000–1:117000), X-linked, multisystemic lysosomal storage disorder caused by deficient activity of the alpha-galactosidase (α-GAL) enzyme. The progressive accumulation of undigested substrate (globotriaosylceramide, GB3) in enzyme deficient cells ends in the multiple organ failure that characterizes classic AFD [1] or, vice versa, in preferential organs such as heart or kidney, in the late-onset variants of the disease [2]. Diagnostic criteria, including GI involvement, and therapeutic goals for AFD are well-coded [3]. The evaluation of GI dysfunction is based upon symptoms that occur in more than 50% of patients [4]. This “clinical” prevalence may not correspond to the specific diagnosis of Fabry gastrointestinal disease because symptoms are non-specific and similar to those caused by common diseases that are highly prevalent in the general population; in addition, biopsies are not routinely performed and, even when performed, do not target the search of GB3 accumulation [5]. In patients with classic AFD, gastrointestinal disturbances may severely impair quality of life. GI symptoms, particularly those related to slowed gastric emptying and those due to increased intestinal motility, can be the first, and often unrecognized, clinical manifestations of classic AFD [6–8]. However, non-specific GI disturbances may also occur in patients with late-onset variants of AFD. GI symptoms in patients with AFD are imputed to the syndrome, irrespective of the demonstration of the GB3 accumulation in the GI cells.

While GI symptoms lack specificity, the pathological findings are specific and tissue biopsy may provide an unequivocal diagnosis [6]. We investigated the pathologic substrate of AFD gastropathy in classic vs. late-onset AFD vs. controls.

## Methods

The clinical series is constituted of 6 unrelated probands, including two classic AFD [GLA p.(Ser401*) and p.(Ala352Asp)], two late-onset AFD [GLA p.(Asn215Ser)] and two control cases [carriers of the benign variant GLA p.(Asp313Tyr]. The two patients with classic AFD as well as those with the late-onset AFD were being treated with enzyme replacement therapy (ERT), while control carriers of the p.(Asp313Tyr) were not.

All patients had long-lasting GI disturbances that were poorly controlled with medications commonly used for such symptoms (Supplemental Table).

GI manifestations in the two male patients with classical AFD (27 and 22 years) started in early infancy, with nausea and vomiting, epigastric pain and alvus disturbances in addition to fever crisis, headache, chronic burning, and acral pain. The symptoms were poorly controlled with high dose painkillers and required multiple hospital admissions, especially in infancy.

The two patients with late-onset AFD p.(Asn215Ser) have typical prominent cardiac manifestations with myocardial GB3 accumulation. One is a 17-year-old boy who developed severe GI symptoms consisting of epigastric pain, vomiting and weight loss (>10Kg in a few months) after the death of his maternal grandmother. The second patient is a 51-year-old female complaining of epigastric pain, heartburn, and nausea since age 46 years.

The two control cases, a 50-year-old female and an 81-year-old male, both carriers of the benign variant GLA p.(Asp313Tyr), complained of epigastralgia, nausea, vomiting, early satiety, and constipation.

We performed esophagogastroduodenoscopy (EGD) with gastric biopsy to identify the causes of the GI symptoms. Gastric biopsy samples were processed for routine histopathology and electron microscopy, and immune-stained with anti-GB3 antibodies [9].

## Results

EGD demonstrated gastroesophageal reflux disease in the female carrier of late-onset AFD p.(Asn215Ser) and in both carriers of the benign p.(Asp313Tyr) GLA variant. The endoscopy of the 51-year-old female patient with late-onset AFD also showed grade B esophagitis, foveolar hyperplasia, and fundic gland polyps.

**Fig. 1:**
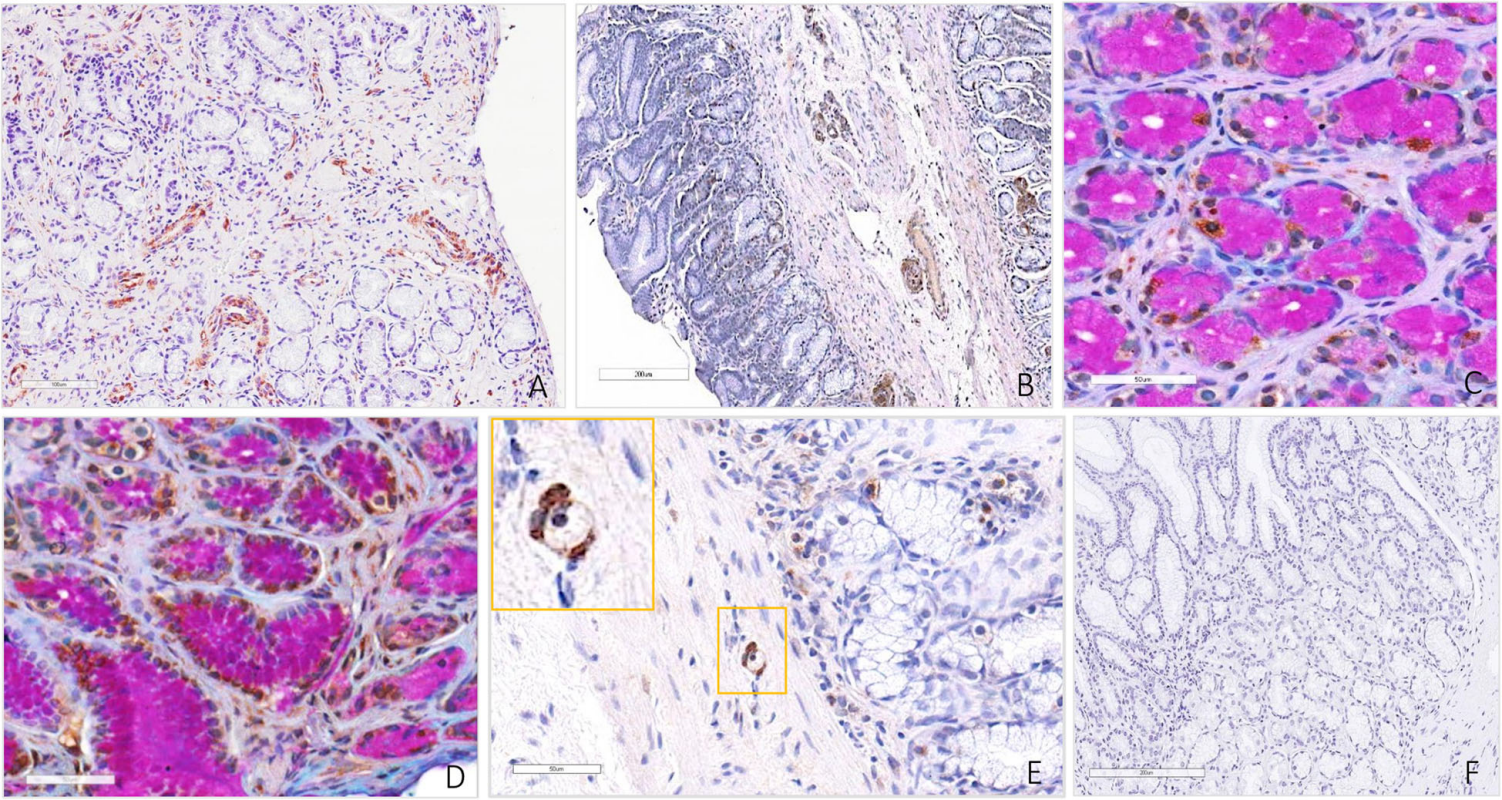
**a** and **b**. The light microscopy view of anti-GB3 immunostain of the gastric biopsy sample of the two patients with classical AFD. **c** and **d**. Alcian PAS and immunostain of the gastric biopsy with positive immune-reaction (brown) to anti-GB3 antibodies. **e** Patient 1. Anti-GB3 immunostain showing a ganglion cell (squared by the yellow box and enlarged in the inset) of the submucosal plexus with foam cytoplasm only mildly immunoreactive with anti-GB3 antibodies, surrounded by intensely immunostained neurilemmal cells. **f** Light microscopy view of negative anti-GB3 immunostain of a sample of the gastric biopsy from patient 5. Identical results were obtained in patients 3, 4, and 6 (Bars provide the original magnification scale)

**Fig. 2:**
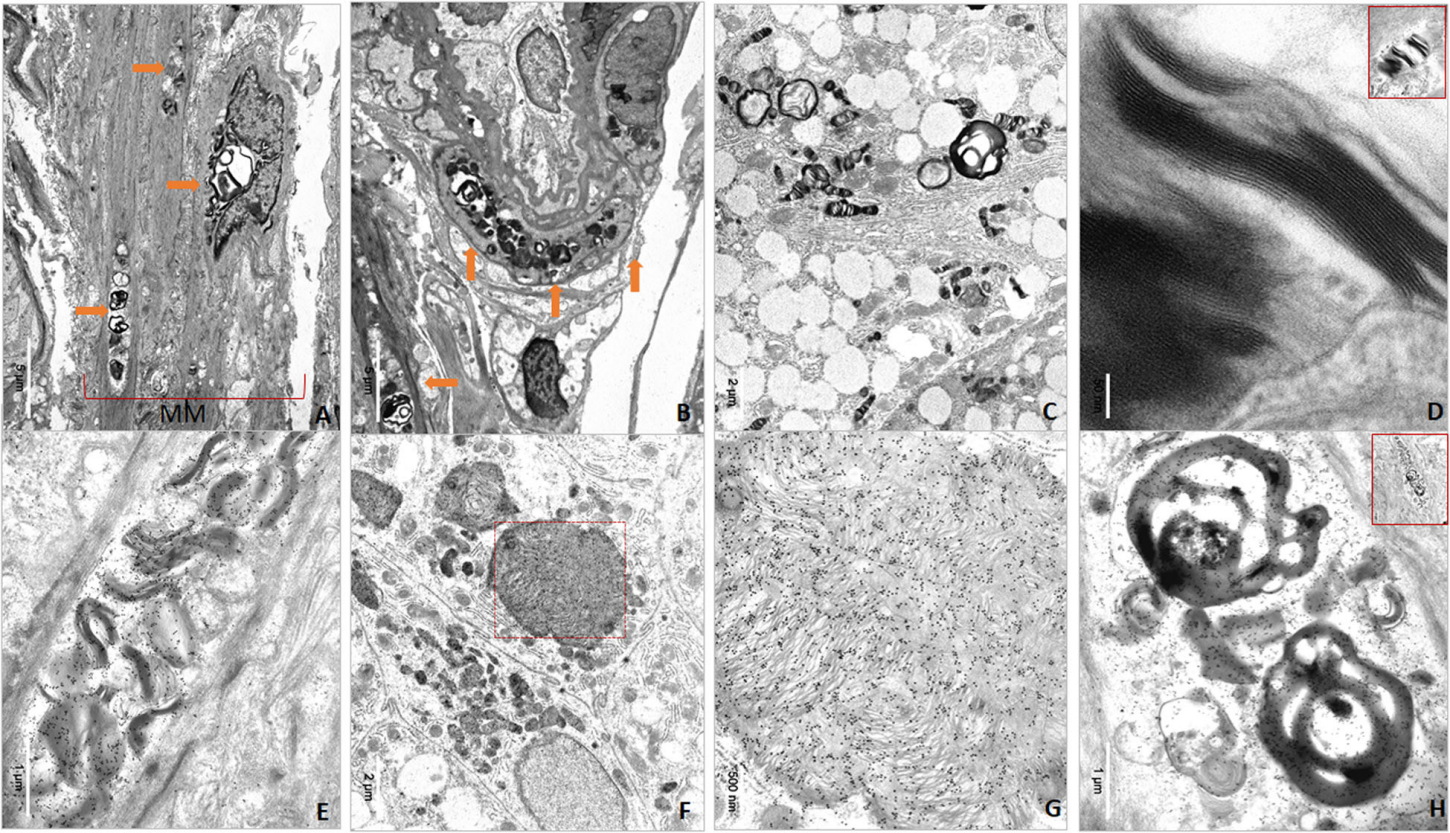
Electron and immune-electron microscopy of the gastric biopsy. **a** Muscularis mucosae showing typical osmiophilic intracytoplasmic bodies (arrows); **b** Vascular smooth muscle cell with GB3 accumulation (arrows); **c** Epithelial cell with multiple osmiophilic intracytoplasmic, lamellar bodies; **d** High magnification view demonstrating the typical parallel stacked lamellae with a zebra-like pattern that characterizes the GB3 bodies; the red-squared figure (upper right corner) shows the original body at low magnification. **e** Immunoelectron micrograph demonstrating the specific reaction of anti-GB3 antibodies in the osmiophilic bodies’ accumulations; **f** Epithelial cells of the gastric mucosae showing intra-cytoplasmic accumulation of GB3 specifically immunostained with anti-GB3 antibodies; **g** The red-squared area in (**f**) is shown at higher magnification in (**g**); **h** Immunoelectron micrograph demonstrating the specific reaction of GB3 antibodies in a non-vascular SMC, a fine extension of the muscularis mucosae extending up between the glands. The red-squared figure (upper right corner) shows the original cell at low magnification

In the 2 patients with classic AFD, the immunohistochemical study with anti-GB3 antibodies showed specific immunostain of endothelial and vascular smooth muscle cells (SMC), pericytes, nerve cells, interstitial mesenchymal cells, and epithelial cells (Fig. 1a-e). The ultrastructural study demonstrated the typical lamellar osmiophilic bodies in the cytoplasms of the SMC of the muscularis mucosae, vascular and non-vascular SMCs, nerve cells and gastric epithelia (Fig. 2a-d). The electron microscopy study confirmed the specific anti-GB3 immunostain (Fig. 2f-h). We diagnosed Fabry’s gastropathy with the involvement of vascular, nervous and epithelial cell compartments.

Vice versa, the gastric biopsies from the two cases with late-onset AFD caused by the p.(Asn215Ser) mutation, similar to the control cases, did not show GB3 accumulation (Fig. 1f). In these 4 patients, GI symptoms were attributable to gastric comorbidities. In all 6 patients, Helicobacter Pylori tested negative.

## Discussion

The gastric mucosa of patients with classic AFD demonstrates substrate accumulation in epithelial, vascular, nervous and interstitial cells. These results are consistent with those observed in other organs/tissues in patients with classic AFD heart: myocytes, vessels and nerves, kidney: podocytes, tubular cells, vessels and nerves [10]. A few past pathology studies of duodenal, jejunal and rectal samples demonstrated a normal villous pattern and luxol-fast blue positive “foamy” cell deposits and “electron dense, intra-lysosomal zebra-like bodies in the vascular endothelial and perithelial cells and in the cytoplasm of the small unmyelinated neurons, and perineurial cells” [10]. Similar findings were described in bowel and rectal biopsies [11, 12].

The main achievement of our study is the demonstration of GB3 accumulation in epithelial, vascular and nerve cells of patients with classical AFD patients complaining of gastric symptoms and the exclusion of GB3 accumulation in the gastric mucosa of patients with later onset AFD p.(Asn215Ser) as well as in control carriers of the benign variant [p.(Asp313Tyr)]. In both late-onset and controls, GI symptoms are not explained by the GB3 accumulation but rather by common adult-age comorbidities. Although myenteric plexus is not sampled by gastric biopsies, gastric samples contain nerve cells and fibers, and, depending on the depth of the samples, ganglion cells of the submucosal plexus (Fig. 1e). The precise diagnosis of Fabry gastropathy is easy to replicate. If systematically investigated, it could represent a possible source of information for measuring ERT effectiveness, e.g. targeting the clearance of endothelial cells, as shown in multiple cell types of kidney biopsies after ERT [13]. However, baseline (before ERT) gastric biopsies were not available in our patients, and we can only infer that GB3 is present in gastric cells of ERT-treated patients with classical AFD. Implementation of the gastric biopsy could contribute, at least partially, to elucidate the origin of symptoms and provide data on the actual burden of Fabry-specific gastric disease particularly in patients with the classical phenotype. Past available evidence was based solely on symptoms. This approach cannot distinguish Fabry gastropathy from other more common forms of gastric diseases or from gastropathies in which AFD may have a contributory role.

In conclusion, Fabry gastropathy, while not life-threatening, definitely impairs quality of life and requires frequent gastroenterology visits, with variable and often frustrating clinical benefits. A systematic study of the pathological bases of Fabry gastropathy could help in understanding how to better control symptoms and evaluate the efficacy of disease-specific treatments. Further studies are needed to expand this observation and to assign a precise role of GB3 accumulation in the origin of gastric symptoms in AFD.

## Supplementary information

**Additional file 1 Supplementary Table** – Patients’ clinical features.

## Data Availability

Data described in this paper are available c/o Center for Inherited Cardiovascular Diseases, Fondazione IRCCS Policlinico San Matteo, Pavia, Italy .

## Abbreviations

AFD: Anderson-Fabry Disease
EGD: Esophagogastroduodenoscopy
ERT: Enzyme Replacement Therapy
GI: Gastrointestinal
GB3: Globotriaosylceramide
PAS: Periodic Acid-Schiff
SMC: Smooth Muscle Cells

## References

[1] Germain DP (2010). Fabry disease. Orphanet J Rare Dis.

[2] Oder D, Liu D, Hu K, et al. α-Galactosidase a genotype N215S induces a specific cardiac variant of Fabry disease. Circ Cardiovasc Genet. 2017;10(5):e001691. 10.1161/CIRCGENETICS.116.001691. 10.1161/CIRCGENETICS.116.001691 29018006

[3] Wanner C, Arad M, Baron R (2018). European expert consensus statement on therapeutic goals in Fabry disease. Mol Genet Metab.

[4] Hoffmann B, Schwarz M, Mehta A, Keshav S (2007). Fabry outcome survey European investigators. Gastrointestinal symptoms in 342 patients with Fabry disease: prevalence and response to enzyme replacement therapy. Clin Gastroenterol Hepatol.

[5] Hilz MJ, Arbustini E, Dagna L (2018). Non-specific gastrointestinal features: could it be Fabry disease?. Dig Liver Dis.

[6] Zar-Kessler C, Karaa A, Sims KB, Clarke V, Kuo B (2016). Understanding the gastrointestinal manifestations of Fabry disease: promoting prompt diagnosis. Therap Adv Gastroenterol.

[7] Politei J, Thurberg BL, Wallace E (2016). Gastrointestinal involvement in Fabry disease. So important, yet often neglected. Clin Genet.

[8] Marchesoni CL, Roa N, Pardal AM (2010). Misdiagnosis in Fabry disease. J Pediatr.

[9] Favalli V, Disabella E, Molinaro M (2016). Genetic screening of Anderson-Fabry disease in probands referred from multispeciality clinics. J Am Coll Cardiol.

[10] Sheth KJ, Werlin SL, Freeman ME (1981). Gastrointestinal structure and function in Fabry’s disease. Am J Gastroenterol.

[11] O’Brien BD, Shnitka TK, McDougall R (1982). Pathophysiologic and ultrastructural basis for intestinal symptoms in Fabry’s disease. Gastroenterology..

[12] Schiffmann R, Rapkiewicz A, Abu-Asab M (2006). Pathological findings in a patient with Fabry disease who died after 2.5 years of enzyme replacement. Virchows Arch.

[13] Thurberg BL, Rennke H, Colvin RB (2002). Globotriaosylceramide accumulation in the Fabry kidney is cleared from multiple cell types after enzyme replacement therapy. Kidney Int.

