# Supplementary material for: Pathologic substrate of gastropathy in Anderson-Fabry disease
Source: Orphanet J Rare Dis. 2020 Jun 22;15:156. doi: 10.1186/s13023-020-01436-2 (PMC7310490; doi:10.1186/s13023-020-01436-2)
Supplement: Supplementary file 1 — Additional file 1 Supplementary Table – Patients’ clinical features. [file 13023_2020_1436_MOESM1_ESM.docx]

Supplementary Table – Patients’ clinical features

| # | Age | Sex | *GLA* variant | inherited/  de novo | Gene Variant (acmg class) | Facial Traits | Heart  LV wall thickness | Brain  involvement | Kidney | Skin  Angiokeratomas (AGK) | Eye | Acral  Symptoms | Gastropathy/  Gastric Biopsy GB3 accumulation | risk factors; comorbidities |
| --- | --- | --- | --- | --- | --- | --- | --- | --- | --- | --- | --- | --- | --- | --- |
| 1 | 27 | M | p.(Ala352Asp) | *De novo* | Pathogenic | Pseudo-acro-megalic facial traits, peri-orbital fullness, bushy eyebrows, recessed forehead, prominent nasal angle. | 11mm | Negative Brain MR. | Intermittent micro-albuminuria | AGK: Peri-umbilical,peri-labial, peri-ungueal, bathing suit areas. Fordyce type case #1. | CV | Severe in infancy and persistent in adult age | Fabry Gastropathy, GB3 positive | Anxiety, Depression |
| 2 | 22 | M | p.(Ser401*) | Inherited | Pathogenic |  | 10mm | Negative Brain MR. |  |  | CV |  | Fabry Gastropathy, GB3 positive; GERD | Psychosis |
| 3 | 51 | F | p.(Asn215Ser) | Inherited | Pathogenic | None | 12-13 mm | Negative Brain MR. | No | No AGK. A few thoracic cherry angiomas. | No | No | GERD with Esophagitis. GB3 negative | Hypertension |
| 4 | 17 | M | p.(Asn215Ser) | Inherited | Pathogenic | None | 10 mm | No Brain MR. | No | No AGK. | No | No | Vomiting and weight loss. Nonsignificant macro and microscopic abnormalities.  GB3 negative | Anxiety after a family mourning |
| 5 | 50 | F | p.(Asp313Tyr) | Inherited | VUS | None | 11 mm | Migraine with aura; brain MR  showing WMLs | Intermittent proteinuria | No AGK; Cherry angiomas. | No | Distal/acral parastesias:> with cold. Raynaud. | GERD with  Esophagitis grade B. GB3 negative. | Hypertension; abdominal pain🡪 laparoscopic excision of right ovarian cyst |
| 6 | 81 | M | p.(Asp313Tyr) | Inherited | VUS | None | 16mm | Stroke | Proteinuria,  multiple renal cysts  (PKD excluded) | No AGK; Multiple cherry angiomas | No | Pain: Small joints. Paresthesia: hands and feet. | GERD with  Esophagitis grade B. GB3 negative. Diverticulosis | Hypertension; DM2;  Hyper-TG;  Hyper-cholesterolemia |

Abbreviations: ACMG = American College of Medical Genetics; AGK = Angiokeratomas; GI = Gastrointestinal; CV = Cornea Verticillata; DM2 = Diabetes Mellitus type 2; GERD = Gastroesophageal reflux disease; LV = Left Ventricle; MR = Magnetic Resonance; PKD = Polycystic kidney disease; TG = Triglyceridemia; WMLs = White Matter Lesions.
